# Visualization of intestinal infections with astro- and sapovirus in mink (*Neovison vison*) kits by *in situ* hybridization

**DOI:** 10.1093/femsmc/xtab005

**Published:** 2021-04-16

**Authors:** Julie Melsted Birch, Mikael Leijon, Søren Saxmose Nielsen, Tina Struve, Henrik Elvang Jensen

**Affiliations:** Section for Pathobiological Sciences, Department of Veterinary and Animal Sciences, University of Copenhagen, 1870 Frederiksberg C, Denmark; Kopenhagen Fur, 2600 Glostrup, Denmark; Department of Microbiology, National Veterinary Institute, 751 89 Uppsala, Sweden; Section for Animal Welfare and Disease Control, Department of Veterinary and Animal Sciences, University of Copenhagen, 1870 Frederiksberg C, Denmark; Kopenhagen Fur, 2600 Glostrup, Denmark; Section for Pathobiological Sciences, Department of Veterinary and Animal Sciences, University of Copenhagen, 1870 Frederiksberg C, Denmark

**Keywords:** mink, sticky kits, diarrhea, intestine, virus, *in situ* hybridization

## Abstract

Clarification of the infection microbiology remains a challenge in the pre-weaning diarrhea (PWD) syndrome in farmed mink (*Neovison vison*). Duodenal, jejunal and colon sections from 36 mink kits with PWD were systematically examined by chromogen *in situ* hybridization targeting two incriminated viruses: mink astrovirus and mink sapovirus. Using the RNAscope^®^ 2.5 HD Duplex Assay, astrovirus and sapovirus were visualized and simultaneously demonstrated in the gut tissue. Both viruses infect enterocytes in the small intestine with a specific localization pattern; astrovirus affects the two apical thirds of the villi, whereas sapovirus generally affects the basal parts of the villi. Furthermore, we demonstrated that astrovirus in mink does not target the goblet cells. This is the first time astro- and calicivirus have been visualized in mink kit gut tissue, and these findings might be important in clarification of the impact of these viruses in the PWD syndrome.

## INTRODUCTION

The aetiology of pre-weaning diarrhea (PWD) in mink kits has been of concern for decades due to the negative impact on animal health, antibiotic consumption and losses caused by recurring disease outbreaks. Suckling kits between 1 and 4 weeks of age are affected manifesting as diarrhea and a concurrent skin exudation, and are referred to as ‘sticky kits’ or ‘wet kits’ among mink farmers. The syndrome is multifactorial and several risk factors concerning parity profile, management and feeding of the dams have been found (Chriél 1997; Møller and Chriél 2000; Møller 2004; Birch *et al*. 2017, 2018a). The outbreak patterns on farms suggest a contagious origin. However, research aiming to elucidate the role of bacterial and viral infections in the PWD syndrome suggests an interplay between several bacteria and viruses since several of the incriminated microorganisms also have been identified in healthy mink kits (Jørgensen, Scheutz and Strandbygaard 1996; Vulfson *et al*. 2001, 2003; Guardabassi *et al*. 2012; Birch *et al*. 2018b). Research in the viral aetiology of PWD has shown that astrovirus was significantly associated with PWD in mink (Englund *et al*. 2002), and mink astrovirus (MiAstV) from intestinal contents was later characterized (Mittelholzer *et al*. 2003). Other viruses such as calicivirus have also been detected in the mink gut (Svansson 1991; Jørgensen, Scheutz and Strandbygaard 1996). Guo, Evermann and Saif (2001a) detected and characterized mink enteric calicivirus in clinically normal and diarrheic mink, and calicivirus detected by electron microscopy has been shown to be associated to PWD, although to a lesser extent than astrovirus (Englund *et al*. 2002). In recent studies, we have taken advantage of next-generation sequencing (NGS) to show that besides MiAstV, also calicivirus, of the genus sapovirus (SaV), seems to be associated with PWD (Birch *et al*. 2018b).

Previously, we have detected both astrovirus and sapovirus sequences on the same farm (Birch *et al*. 2019). This has led to questions concerning which parts of the gastrointestinal (GI) tract actually are infected by the two viruses, how the distribution pattern is and which cell populations are infected. In this study, our objective was to study the colocalization of astrovirus and sapovirus in enterocytes and goblet cells in different parts of the gut in mink kits with PWD using branched DNA *in situ* hybridization.

## MATERIALS AND METHODS

### Animals and study design

On a Danish mink farm, 36 mink kits, 6–21 days old, with manifestations consistent with PWD were collected during a period of four consecutive days in May 2017. The diarrheic mink kits were all from different litters and met the inclusion criteria by the presence of runny, loose or liquid feces or if the feces had a white or beige colour as a sign of undigested milk. Moreover, the mink kits showed varying degrees of red swollen anus, dirty perineal region and skin exudation (Birch *et al*. 2018a). After euthanization by a stroke to the head followed by decapitation, necropsy was done immediately afterwards. Aseptically, the GI tract was evacuated and a piece of the proximal duodenum, mid gut (jejunum) and distal colon was placed and fixed in 10% formalin. The animals used in this project were solely euthanized for collection of their tissues/organs and thereby in accordance with the Danish Order regarding animal experimentation §2, article1; hence, approval from ethics committees was not required. Euthanasia was carried out by an authorized veterinarian in accordance with the European Council Regulation (EC) No. 1099/2009 regarding protection of animals at the time of killing.

### *In situ* hybridization

Standard procedures for dehydration and paraffin embedding were applied (Spencer and Bancroft 2008), after which 3-µm-thick sections were cut on a microtome and mounted on SuperFrost Plus slides (vWR, Radnor, PA). On a CLC genomic workbench version 11 (CLC GW, Qiagen, Hilden, Germany), consensus sequences for mink astrovirus and sapovirus were generated based on NGS results from two previous studies (Birch *et al*. 2018b, 2019). Based on the consensus sequences, gene-specific probe pairs targeting astrovirus (channel 1) and sapovirus (channel 2) were provided for the RNAscope^®^ 2.5 HD Duplex Assay (ACD, Bio-Techne, MN, USA). Duplex probe sets for positive controls were designed based on two commonly used housekeeping genes published for mink in the National Center for Biotechnology Information (NCBI): Gapdh mRNA (GenBank: KM025344) and beta-actin (Actb) mRNA (GenBank: EU046492.1), which previously have been used as reference genes (Zhang *et al*. 2009; Bowman and Rose 2017). The duplex positive control was designed with the Gapdh probe in channel 1 and the Actb probe in channel 2 (ACD, Bio-Techne, MN, USA). The duplex positive control probe and RNAscope^®^ 2-Plex Negative Control Probe (ACD, Bio-Techne, MN, USA) were included in each assay. In addition, positive controls including astrovirus and sapovirus as well as negative control tissue without virus (foetal gut tissue) were included in each run. After hybridization, amplification and detection of the duplex signal, green and red for channels 1 and 2, respectively, the slides were counterstained with Gills Hematoxylin No. 1 (Sigma-Aldrich, St Louis, MO, USA) and mounted with Vectamount (Vector Laboratories, Burlingame, CA, USA). By this *in situ* hybridization method, each single target molecule (e.g. from a virus or a housekeeping gene) was visualized as a consequence of the branched amplification. In order to elucidate if astrovirus targets the intestinal goblet cells, periodic acid solution (PAS) with specificity for mucins was applied. On one random astrovirus positive sample, a PAS-staining step (Myers, Fredenburgh and Grizzle 2008) was added after the green and red detection step and prior to the counterstaining.

### Histological assessment of *in situ* hybridization signals

From each animal, cross-sections from the duodenum, jejunum and colon were examined by light microscopy at 20× magnification. The load (i.e. burden of infection) of astro- and sapovirus in duodenum and jejunum was individually assessed using the following approach: infected villi (VIL) were scored as ‘0’ (0%), ‘1+’ (<5%), ‘2+’ (5–50%) or ‘3+’ (50–100%). Second, the number of virus positive enterocytes per affected villus (VPE/VIL) was scored as ‘0’ (0), ‘1+’ (<5), ‘2+’ (5–10) or ‘3+’ (>10). Analogously, the virus load in the colon sections was assessed as % infected crypts (CRYPT), and affected enterocytes/infected crypt (VPE/CRYPT). The locations of virus replication in the duodenum and jejunum sections were assessed if it was present in the (i) crypts, (ii) basis of villi (basal third), (iii) middle of villi (central third) or (iv) top of villi (apical third).

### Statistics

A common measure of the burden of infection, the virus load score (VLS), for each duodenal and jejunal section was generated by adding VIL and VPE/VIL, and CRYPT and VPE/CRYPT for colon sections. These scores were summarized using summary statistics for the positive sections. The resulting ordinal scores were compared using the Wilcoxon rank-sum test in R v. 4.0.2 (R Core Team 2020). Subsequently, the proportion of positive sites for each location (crypt, basis of villi, middle of villi, top of villi) for duodenal and jejunal sections separately was plotted and compared using a quasibinomial model to compare the presence of each virus at different sites and intestinal sections. Pairwise comparisons of the resulting estimates were done and corrected using Tukey's procedure for post-hoc assessment using the multcomp package in R (Hothorn, Bretz and Westfall 2008). The dataset is available in Table S1 (Supporting Information).

## RESULTS

Astrovirus was detected in at least one of the three gut sections in 33/36 (91.7%) of the animals, whereas sapovirus was detected in 18/36 (50%) of the animals. Coinfection with both astro- and sapovirus was observed in 15/36 (41.7%) of the animals. Astrovirus was exclusively found in 18/36 (50%) of the animals, and in 3/36 (8.3%) of the animals exclusively sapovirus was detected, whereas none of the animals were negative for both astro- and sapovirus. Summary statistics of the VLS are shown in Table 1.

**Table 1.** VLS of astro- and sapovirus detected by *in situ* hybridization.

	Median	q1	q3	Min.	Max.
All samples					
**Astrovirus** (*n* = 36)					
Duodenum	4	2	5	0	6
Jejunum	5	2	6	0	6
Colon	0	0	2	0	5
**Sapovirus** (*n* = 36)					
Duodenum	0	0	5	0	6
Jejunum	2	0	5	0	6
Colon	0	0	0	0	2
Virus positive samples					
**Astrovirus** (*n* = 33)					
Duodenum	4	3	5	0	6
Jejunum	5	3	6	0	6
Colon	0	0	2	0	5
**Sapovirus** (*n* = 18)					
Duodenum	5	3	6	0	6
Jejunum	6	5	6	3	6
Colon	0	0	0	0	2

VLS was generated by adding VIL and VPE/VIL, where VIL was infected villi [‘0’ (0%), ‘1’ (<5%), ‘2’ (5–50%) and ‘3’ (50–100%)] and VPE/VIL was virus positive enterocytes per villi [‘0’ (0), ‘1’ (<5), ‘2’ (5–10), ‘3’ (>10)]. q1; first quartile, q3: third quartile.

Among astrovirus positive animals (*n* = 33), virus was detected in higher loads (VLS) in duodenal and jejunal sections compared with colon sections (*P* < 0.001 and *P* < 0.001, respectively), whereas there was no significant difference in VLS between the duodenal and jejunal sections. Likewise, among sapovirus positive animals (*n* = 18), VLS of sapovirus also was higher in duodenal and jejunal sections compared with colon sections (*P* < 0.001 and *P* < 0.001, respectively). In addition, sapovirus load score was higher in jejunum compared with duodenum among sapovirus positive samples (*P* < 0.05).

**Figure 1.**
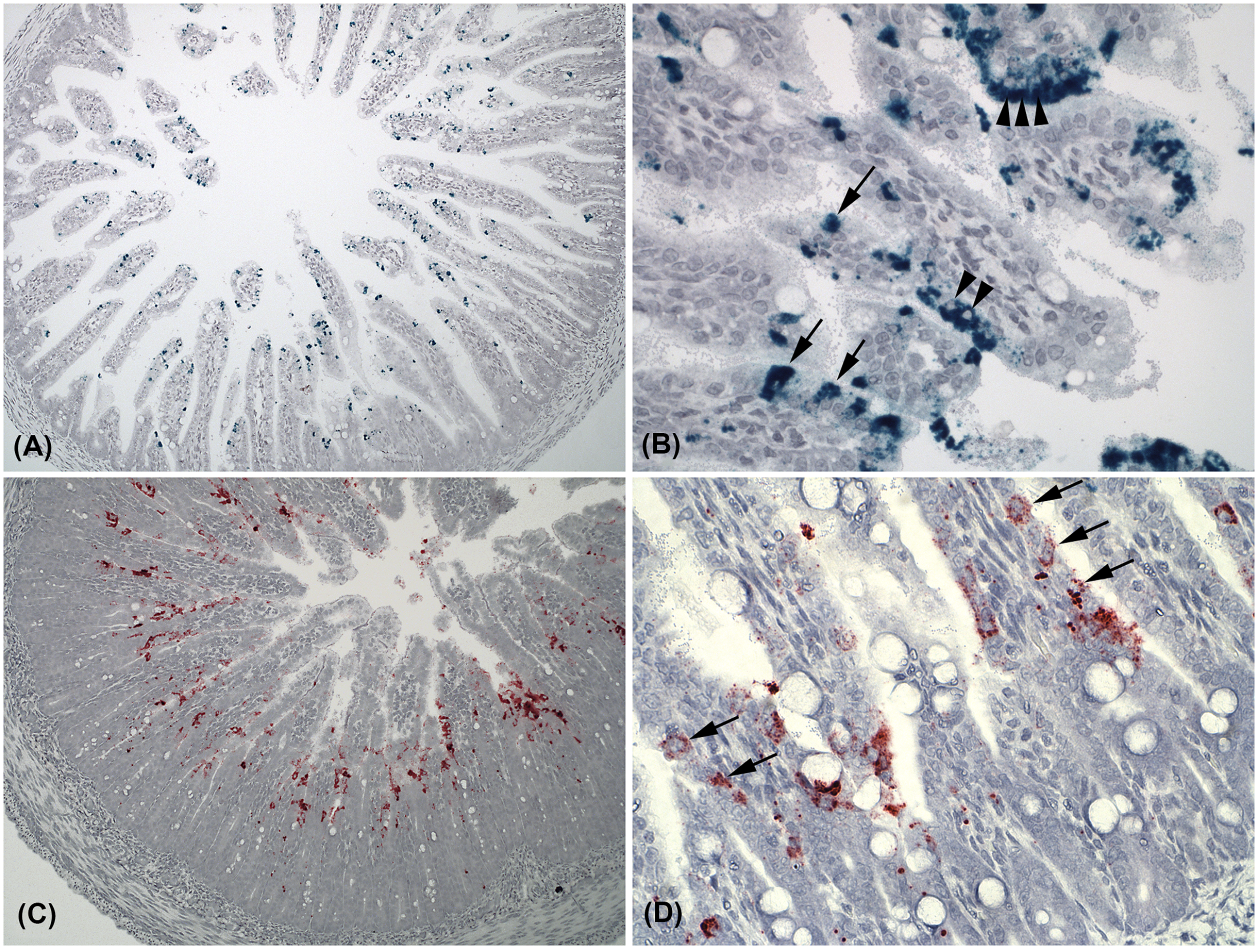
Photomicrographs of *in situ* hybridization targeting astro- and sapovirus in intestinal samples from mink kits with PWD. **(A)** Transverse section of a jejunum with astrovirus (green) infection (×10). **(B)** Astrovirus infection at the tips of villi in jejunum. Note that single enterocytes contain very high loads of virus next to not infected neighbour cells (arrows), and that replication is located to the cytoplasm and not the nucleus (small arrowheads) (×40). **(C)** Jejunal transverse section with sapovirus (red) infection (×10). **(D)** Higher magnification (×40) of sapovirus infection at the base of the villi in the duodenum. Replication of sapovirus is located to the cytoplasms of infected enterocytes (arrows).

**Figure 2.**
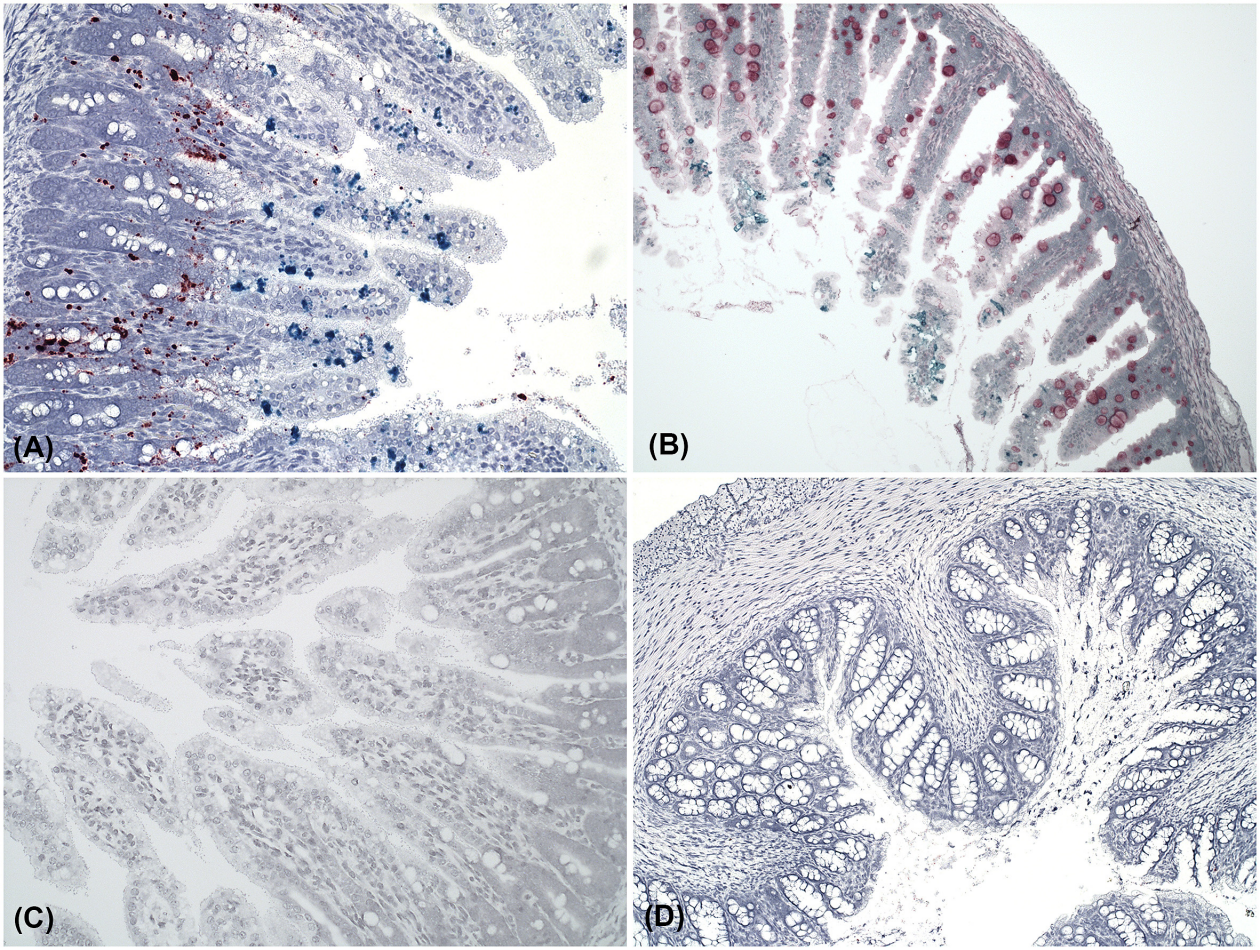
Photomicrographs of*in situ* hybridization targeting astro- and sapovirus in intestinal samples from mink kits with PWD. **(A)** Coinfection of both astro- (green) and sapovirus (red) in a transverse section of jejunum. Notice the sharp distinction of the location of the two viruses (×20). **(B)** An astrovirus positive duodenal section. PAS staining added to the duplex *in situ* hybridization assay before counterstaining. Note that astrovirus positive enterocytes are positioned apart from the goblet cells containing mucin (red) (×20). **(C)** Jejunum, negative control for both green and red channels (×20). **(D)** A colon section negative for both astro- and sapovirus (×10).

In the small intestine, it was evident that astrovirus primarily affected the apical 2/3 of the villi (Fig. 1A and B). In comparison, sapovirus was typically found in the basal enterocytes of the villi (Fig. 1C and D and Fig. 2A) and also in some crypts, which was not the case for astrovirus in any of the samples.

In approximately half of the sections containing sapovirus, virus extended to more apical parts of the villi. However, these were typically sections exclusively containing sapovirus (Fig. 1C). In all astro- and sapovirus positive samples (*n* = 15), astrovirus was located apical to sapovirus infected enterocytes (Fig. 2A). Addition of a PAS staining step in an astrovirus positive sample showed no merging between astrovirus positive enterocytes and goblet cells (Fig. 2B). Photomicrographs of a 2-plex negative control and a virus negative colon section are shown in Fig. 2C and D.

**Figure 3.**
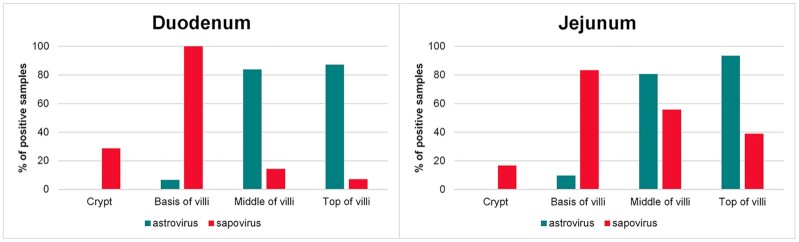
Locations of astro- and sapovirus in the small intestinal sections.

Locations of the viruses are displayed in Fig. 3, with pairwise statistical comparisons of astro- and sapovirus locations shown in Table 2.

**Tabel 2.** Pairwise comparison of sapo- and astrovirus locations in the small intestine.

Pairwise comparison	OR	Lower CL (OR)	Upper CL (OR)	*P*-value
**Duodenum**				
Crypt: sapo vs astro	Ref.	n.a.	n.a.	1
Base-villi: sapo vs astro	10.8	4.8	24.2	0.14
Mid-villi: sapo vs astro	0.023	0.010	0.051	<0.001
Top-villi: sapo vs astro	0.010	0.003	0.028	<0.01
**Jejunum**				
Crypt: sapo vs astro	Ref.	n.a.	n.a.	1
Base-villi: sapo vs astro	7.9	3.9	15.7	0.14
Mid-villi: sapo vs astro	0.169	0.101	0.28	<0.05
Top-villi: sapo vs astro	0.058	0.032	0.106	<0.0001

n.a.: not applicable; OR: odds ratio; CL: confidence limit.

## DISCUSSION

For the first time, we have visualized the location of astrovirus and sapovirus infections in intestines of mink kits with PWD. Furthermore, we have demonstrated coinfection with these viruses in the same tissues by use of the RNAscope *in situ* hybridization duplex assay. Astrovirus was most frequently detected (92% of the animals) compared with sapovirus, which was observed in 50% of the study group. These findings correspond well with other studies in which these two viruses have been found to increase the risk of PWD (Englund *et al*. 2002; Birch *et al*. 2018b). The load of both astro- and sapovirus was significantly higher in the small intestine (duodenum and jejunum) than in the colon, which indicates that potentially harmful effects of the viruses are also predominantly located to the small intestine. However, the astrovirus infection extended to enterocytes of the colon in some cases, and here the crypts were deeper, which suggests that these colon sections might have been collected more proximally compared with the rest of the samples. This is in line with other studies regarding astrovirus infections in humans (Sebire *et al*. 2004), calfs (Woode *et al*. 1984), turkeys (Behling-Kelly *et al*. 2002; Nighot *et al*. 2010) and lambs (Snodgrass *et al*. 1979) in which astroviruses have been located to the small intestine in juvenile individuals. We observed striking differences in the localization between astrovirus and sapovirus, especially in samples with coinfection with the two viruses, which suggests that these two viruses have different cell tropism. Astrovirus mainly affected the two apical thirds of the villi, which is in line with previous studies in lambs and humans (Snodgrass *et al*. 1979; Sebire *et al*. 2004). By contrast, astrovirus in turkeys (TAstV) has been found to affect the basal margins of the villi (Behling-Kelly *et al*. 2002), whereas only the dome epithelium in ileum was affected by astrovirus in calfs (Woode *et al*. 1984). In a recently study, it was shown that murine astrovirus (MuAstV) infects actively secreting goblet cells (Cortez *et al*. 2020). Our studies of astrovirus infection in mink cannot support this finding, as PAS staining of goblet cells and *in situ* hybridization of astrovirus on the same gut sections clearly did not overlap, which suggests that there may be interspecies variation among different mammalian astroviruses with respect to enterocyte type tropism. Replication of both astro- and sapovirus was obviously located to the cytoplasm of the enterocytes (Fig. 1B and D), which is consistent with what is expected for Class IV viruses (Berman 2012). By contrast, we discovered that sapovirus mainly affected the basis of the villi, and in samples with both astrovirus and sapovirus in the same sections, sapovirus was consistently located close to the villus base compared with the apical locations of astrovirus. In some sections, sapovirus was also found in the more distal parts of the villi and in contrast to astrovirus also in the crypts. This finding suggests that astrovirus preferably infects mature enterocytes, whereas sapovirus may prefer replicating in younger enterocytes. Sapovirus has been detected in a variety of species, and phylogenetic studies have shown that variants from species like dogs, sea lions, bats, chimpanzees and rats have genetic similarities to human SaVs (Oka *et al*. 2016), which imply that they probably have somewhat similar properties. In humans, sapovirus is a common cause of self-limiting gastroenteritis especially in young or immunocompromised patients (Kaufman *et al*. 2005; Pietsch and Liebert 2019). Porcine enteric calicivirus (PEC/Cowden strain) associated with diarrhea in swine is a cultivable sapovirus (Guo *et al*. 1999). Our observations of sapovirus affecting the small intestine and not the colon are in accordance with studies on sapovirus in swine where wild-type PECs have been shown to cause mild to severe shortening and blunting of villi in duodenum and jejunum; however, no PEC antigen-positive cells were detected in the colon (Guo *et al*. 2001b). In a prevalence study of noroviruses and sapoviruses in swine, porcine sapovirus (Cowden strain) was found in all age groups, however, highest in post-weaning pigs (83%), whereas nursing pigs had a prevalence of 21% (Wang *et al*. 2006). On the other hand, norovirus was exclusively detected among subclinically infected finisher pigs (Wang *et al*. 2006). Thus, our results also represent an example of sapovirus infection in young individuals. The difference in the location of astrovirus and sapovirus shown in this study may have different impact on the morphological and physiological outcome of the infection. Additional studies of the transcriptomic change in the host GI cell population between single and coinfection with astro- and sapovirus would likely elucidate the impact on the GI tract of these infections.

In conclusion, by use of branched DNA *in situ* hybridization, separate and simultaneous visualization of astro- and sapovirus infection in enterocytes from mink kits with PWD was demonstrated. Both viruses were found in highest loads in the small intestine and generally not in the colon. Our results clearly demonstrate that mink astrovirus and mink sapovirus are able to infect enterocytes of the small intestine in the mink kits in a distinct pattern, which suggests that these viruses by different cell tropisms or predilection sites may have evolved a strategy for co-existence.

## ACKNOWLEDGEMENTS

The authors would like to express their gratitude to laboratory technicians Elisabeth Wairimu Petersen, Betina Gjedsted Andersen and Christina Tirsdal Kjempff for their valuable technical support. Also thanks to Klaus Juel Hansen and Anne Sofie Boyum Johansen for their practical help during material collection.

## Supplementary Material

xtab005_Supplemental_File
